# Long-Term Outcomes in Patients with Intestinal Failure Due to Short Bowel Syndrome and Intestinal Fistula

**DOI:** 10.3390/nu14071449

**Published:** 2022-03-30

**Authors:** Maja Kopczynska, Gordon Carlson, Antje Teubner, Arun Abraham, Michael Taylor, Sorrel T. Burden, Christian L. Hvas, Peter Jepsen, Simon Lal

**Affiliations:** 1 Intestinal Failure Unit, Salford Royal NHS Foundation Trust, Salford M6 8HD, UK; gordon.carlson@nca.nhs.uk (G.C.); antje.teubner@nca.nhs.uk (A.T.); arun.abraham@nca.nhs.uk (A.A.); michael.taylor@nca.nhs.uk (M.T.); sorrel.burden@manchester.ac.uk (S.T.B.); simon.lal@nca.nhs.uk (S.L.); 2 School of Health Sciences, University of Manchester, Manchester M13 9PL, UK; 3 Department of Hepatology and Gastroenterology, Aarhus University Hospital, 8200 Aarhus, Denmark; christian.hvas@auh.rm.dk (C.L.H.); pj@clin.au.dk (P.J.)

**Keywords:** intestinal failure, short bowel syndrome, intestinal fistula, nutritional autonomy, home parenteral nutrition

## Abstract

Short bowel syndrome (SBS) and enterocutaneous or enteroatmospheric fistulas are common indications for home parenteral nutrition (HPN). However, there are few data describing factors influencing surgical decision-making or outcomes particularly following fistula development. We aimed to compare outcomes between patients with SBS and fistulas and explore surgical decision-making. HPN-dependent adults from 2001–2018 at a national reference centre were included in this study. HPN cessation was analysed using death as competing risk. In total, 465 patients (SBS (62%), fistula (38%)) were included, with median HPN dependency of 2.6 years. In total, 203 patients underwent reconstructive surgery; while frailty was the commonest reason for not undergoing surgery (49.2%), 22.7% declined surgery. Overall, 170 ceased HPN, with a probability of 13.8%, 34.1% and 38.3% at 1, 5 and 10 years, respectively. Patients undergoing surgery had higher nutritional autonomy rates (109.8 incidences/1000 patient years) compared to those not undergoing surgery (18.1 incidences/1000 patient years; *p* < 0.001). A total of 295 patients (63.4%) were predicted to cease HPN based on gastrointestinal anatomy but only 162/295 (54.9%) achieved this; those unable to do so were older with a higher comorbidity index. There were no differences in long-term nutritional and survival outcomes or surgical decisions between patients with SBS and fistulas, or between enterocutaneous and enteroatmospheric fistulas. This study represents one of the largest datasets describing the ability of HPN-dependent patients with SBS or fistulas to achieve nutritional autonomy. While reconstructive surgery facilitates HPN cessation, approximately one-fifth of patients declined surgery despite HPN dependency. These data will better inform patient expectation and help plan alternative therapies.

## 1. Introduction

Short bowel syndrome (SBS) following massive intestinal resection, and enterocutaneous fistula (ECF) or enteroatmospheric fistula (EAF) are common causes of intestinal failure (IF) and commonly result in the need for parenteral nutrition (PN) [1]. There are various classifications of IF based on functional, pathophysiological and clinical features: type 1 is an acute, short-term and usually a self-limiting condition; type 2 is a prolonged acute condition, typically in metabolically unstable patients following surgery, which may last for weeks or months; and type 3 IF describes the need, reversible or irreversible, for long-term PN in metabolically stable patients [1]. Patients with SBS or intestinal fistulas requiring prolonged PN typically present acutely with type 2 IF. After a period of inpatient optimisation and metabolic stabilisation using the established ‘Sepsis-Nutrition-Anatomy-Plan’ algorithm [2], they are commonly discharged on home parenteral nutrition (HPN) with type 3 IF. When nutritionally optimised and after a period of recuperation at home, consideration of reconstructive surgery and restoration of bowel continuity may be appropriate [2,3,4]. The decision to perform surgical reconstruction is usually determined by comorbidity, intestinal anatomy, surgical risk and patient choice.

In patients with SBS and intestinal fistulas, the need for permanent PN is determined by the clinical condition, length of remnant small bowel and the type of the digestive circuit after anastomosis [5]. There are three main groups of patients with a short bowel, classified on the basis of the anatomical anastomosis: group 1 (small bowel end-ostomy or fistula); group 2 (jejunocolic anastomosis) and group 3 (jejunoileocolic anastomosis) [6]; for each group, the remnant small bowel length has been shown to be predictive of the ultimate ability to wean PN, since it will provide additional nutrient and fluid absorptive capacity [7]. Thus, surgical reconstruction with restoration of bowel continuity, wherever necessary and possible, to achieve a final small bowel length of ≥115 cm in SBS group 1, ≥60 cm in SBS group 2 and ≥35 cm is predicted to allow patients to achieve nutritional autonomy and HPN cessation [1]. However, there are limited published data relating to factors influencing the decision to perform reconstructive surgery beyond the potential gain in small bowel length that will allow nutritional autonomy. Additionally, there are no published data on the reasons for not proceeding with reconstructive surgery in patients with favourable anatomy, nor on the potential reasons for not achieving nutritional autonomy in those patients with predicted adequate bowel length following surgery. There are, in fact, minimal human data on factors influencing the process of intestinal adaptation following reconstructive surgery, and the vast majority of data have been derived from animal models [8,9]. Furthermore, outcome data on patients with ECF or EAFs are limited primarily to short periods of follow up, with very few studies providing data over periods in excess of 18 months, with the longest reported follow up of only three years [10]. No studies have compared the long-term outcomes of patients with SBS, EAF or ECFs, whether or not they undergo reconstructive surgery.

The aims of this study were therefore to evaluate the likelihood of, and predictors of the restoration of nutritional autonomy in HPN-dependent patients with SBS and intestinal fistulas, to identify the predictors influencing the decision to perform reconstructive surgery and to describe the long-term outcomes of patients with SBS, compared to those with EAFs or ECFs.

## 2. Materials and Methods

### 2.1. Study Cohort

Clinical records of adult patients receiving HPN between 2001 and 2018 cared for at a UK national IF reference centre were reviewed. All consecutive adult patients with SBS defined as a remnant small bowel length of ≤200 cm [1] or ECF/EAFs requiring HPN at our institution were identified from a prospectively maintained database and included in the study. The censoring date for achieving nutritional autonomy was 1 July 2021. Patients were followed up from their first discharge on HPN until death or censoring date. Patients had to be prescribed HPN (parenteral nutrition or fluids and electrolytes) for at least two nights per week. Patients receiving HPN for a diagnosis of underlying malignancy and patients whose care was transferred to another centre were excluded from the study. Participant flow chart is presented in Supplementary Materials Figure S1. This study was approved by a local Research and Innovation Department (Reference No: S21HIP06) as a Service Evaluation and data were managed in accordance with Caldecott Principles to protect patient confidentiality.

### 2.2. Data Collection

Data were collected on patient demographics and clinical characteristics including: start date of HPN, total time requiring HPN, mechanism and severity of IF at the point of discharge as defined by the European Society of Clinical Nutrition and Metabolism (ESPEN) [11], disease leading to IF and comorbidities evaluated using the Charlson Comorbidity Index [12]. Data collected on gastrointestinal anatomy included small bowel length in continuity (measured at surgery and, if not applicable, radiologically) and presence or absence of colon. Where the colon was present, colon length was described according to the method of Cummings et al. [13] and classified as less than 50% or more than 50%, and whether the colon was in continuity. If the patient’s anatomy changed over the study period, both the initial and final anatomy parameters were recorded. Surgical data collected included date and type of surgery (restoration of intestinal continuity, fistula repair and small bowel transplant). Patients were “predicted” to achieve nutritional autonomy based on their final gastrointestinal anatomy: SBS group 1/fistula with small bowel in continuity ≥115 cm; SBS group 2 with small bowel in continuity ≥60 cm and >50% or <50% colon; and SBS group 3 with small bowel in continuity ≥35 cm and intact colon [1]. Reasons for not undergoing reconstructive surgery in patients with favourable gastro-intestinal anatomy (i.e., that the predicted anatomy following reconstruction would result in the relevant small bowel lengths) were also recorded. Patients were followed up every 3 to 6 months, where a nutritional assessment was undertaken. Once nutritionally optimised and where clinically appropriate, reduction in PN was attempted under the monitoring of the multidisciplinary team. If patients remained stable in relation to their hydration (adequate urine output), body weight (no substantial loss of body weight between PN reductions) and serum electrolytes (with or without supplementation), the weaning process was continued until PN cessation. Patients were classified as having achieved nutritional autonomy if they remained off HPN for a period of 12 months.

### 2.3. Statistical Analysis

Categorical variables are described as proportions and continuous variables are described as median (Interquartile range, IQR). Analyses were performed using Chi square for categorical variables and Mann–Whitney U or Kruskal–Wallis for non-parametric continuous variables to compare different groups of subjects. Achieving nutritional autonomy was analysed using death as a competing risk, and cumulative incidence was estimated using the Aalen-Johansen (Aa-J) estimator. This method prevents bias associated with the more commonly used Kaplan–Meier analysis where deaths are censored, which ultimately leads to providing estimates of “probability of HPN dependence in immortal patients” [14]. Fine and Gray regression, which provides sub-distribution hazard ratios taking into account competing risk of death was further performed to assess predictors of achieving nutritional autonomy. Two-tailed *p*-value < 0.05 was considered statistically significant. The statistical software used was R Version 4.0.3 (R Core Team, Vienna, Austria), packages: cmprsk ver. 2.2-10, ComplexHeatmap ver. 2.7.11, UpSetR ver. 1.4.0.

## 3. Results

### 3.1. Patient Demographics

In total, 465 patients were included in the analysis. Median follow-up time was 4.5 years (range 16 days–19.7 years), and the total observation time was 2566.5 patient years. The mechanism of IF at the time of HPN initiation was classified as SBS group 1 in 261 (56.1%) cases, SBS group 2 in 19 (4.1%) cases, SBS group 3 in 5 (1.1%) cases and intestinal fistulas in 180 (38.7%) cases. More patients had ECFs (112/180, 62.2%) than entero-atmospheric fistulas (68/180, 37.8%). The most common diagnosis leading to IF was surgical complications in 198 (42.6%) cases, followed by Crohn’s disease in 118 (25.4%) and mesenteric ischaemia in 107 (23.0%) cases. Patient demographics and clinical characteristics at HPN initiation are summarised in Table 1.

### 3.2. Changes over Study Period

The number of new patients increased per study period from 92 in years 2001–2006 to 148 in years 2007–2012 and 225 in years 2013–2018. Median age at inclusion also increased over the study period (46.5, 55.0 and 61.0 years, respectively, *p* < 0.001), as did the median Charlson Comorbidity Index (score of 1, 2 and 3, respectively, *p* < 0.001).

**Figure 1:**
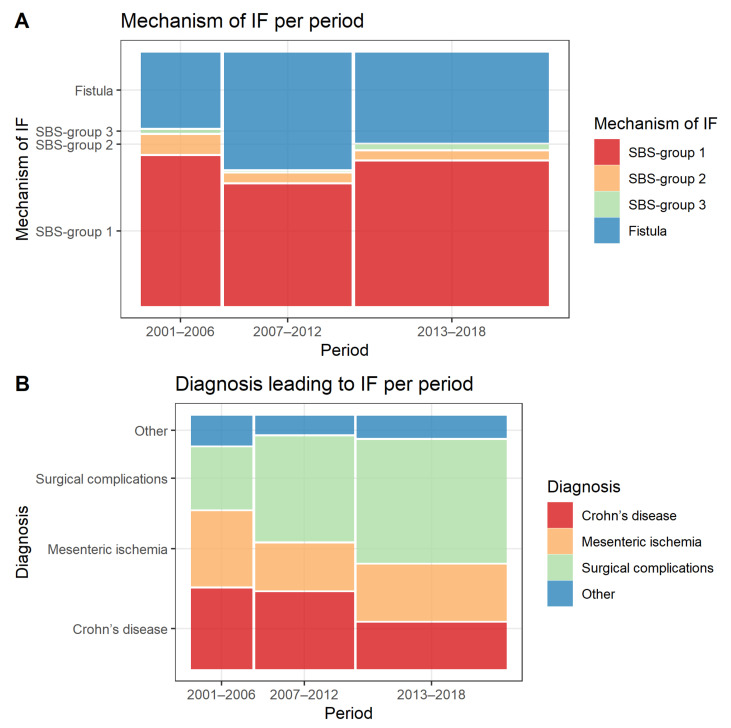
Changes in (**A**) mechanism and (**B**) diagnosis leading to IF over the study period, presented on a mosaic plot where the size of the squares represents the proportion of all included patients. IF, intestinal failure; SBS, Short bowel syndrome.

The proportions of different mechanisms of IF remained stable over the study period with SBS group 1 being the most common mechanism of IF over all time periods, followed by fistulas, SBS group 2 and SBS group 3 (Figure 1A). As regards to the diagnosis leading to IF, there was a decrease in the proportion of patients with Crohn’s disease (32.6%, 31.1% and 18.7%, *p* = 0.005) and an increase in those with surgical complications from other underlying conditions (25.0%, 42.6% and 49.8%, *p* < 0.001) over the study period (Figure 1B).

### 3.3. Reconstructive Surgery

#### 3.3.1. Surgery Time and Types

Two-hundred and three patients underwent reconstructive surgery, with a median time between initiation of HPN and reconstructive surgery of 11.3 months (95% confidence interval (CI) 9.1–14.2). This duration was longer in the later study period (8.3 months vs. 11.4 months vs. 15.6 months), but was similar between SBS and fistulas (11.3 months vs. 11.2 months) and between ECF and EAF (11.2 months vs. 11.2 months). The type of reconstructive surgery involved fistula repair in 70 (34.5%) cases, restoration of continuity in 124 (61.1%) cases and small bowel transplant in 9 (4.4%) cases.

#### 3.3.2. Changes in Anatomy

Patients who underwent transplant surgery were excluded from the analysis of the changes in intestinal anatomy. Median gain in small bowel length after surgery was 60 cm (IQR 0–140 cm). Following surgery, 144/194 (74.2%) patients changed their digestive circuit from end-ostomy or fistula to small bowel in continuity with colon. Anatomy at initiation of HPN, as well as the final anatomy, are summarised in Supplementary Materials Table S1. Median gain in small bowel length after reconstructive surgery was higher for patients with fistula (85 cm, IQR 20–150 cm) compared to patients with SBS (30 cm, IQR 30–116 cm), *p* < 0.001. On the other hand, colon was brought back into continuity in a higher proportion of patients with SBS in comparison to those with fistulas (81.0% vs. 67.0%, *p* = 0.04). Of patients with SBS who underwent surgery, 21.0% had Crohn’s disease, 38.0% mesenteric ischaemia, 31.0% surgical complications and 10% other diagnosis leading to IF. In comparison, of the patients with fistulas undergoing surgery, 30.9% had Crohn’s disease, 2.1% mesenteric ischaemia, 63.8% surgical complications and 3.2% other diagnosis leading to IF.

#### 3.3.3. Potential for Surgery

There were 128 patients who could have potentially undergone surgery to facilitate HPN cessation based on their predicted anatomy (i.e., adequate length of small bowel and/or colon in situ) [1] but did not. The most common reasons for not undergoing surgery were comorbidities or frailty (49.2% cases), followed by patient declining surgery (22.7%), awaiting surgery (14.1%), death prior to intended surgery (12.5%), abdomen deemed too hostile (0.8%) and active Crohn’s disease (0.8%). The causes of death in patients who died prior to intended surgery were: underlying disease (five patients), IF-associated liver disease (IFALD) (three patients), other causes (nine patients) and unknown (one patient).

The proportion of patients who did not undergo surgery despite potentially suitable intestinal anatomy increased over the study period with 13/92 (14.1%) in years 2001–2006, 37/148 (25.0%) in 2007–2012 and 78/225 (34.7%) in 2013–2018. The reasons for patients not to undergo surgery also changed over the time periods, with an increase in the proportion of patients with comorbidities preventing surgery (15.4%, 56.8% and 51.3%, *p* = 0.03) and decrease in the proportion of patients who died prior to intended surgery (46.2%, 16.2% and 5.1%, *p* < 0.001). For patients who declined surgery, the median age was 60 years (IQR 53–73) and Charlson Comorbidity Index was 3 (IQR 1–4), which was not statistically different to patients who did not undergo surgery for other reasons, median age 64 years (IQR 53–72), *p* = 0.87 and median Charlson Comorbidity Index 3 (IQR 2–4), *p* = 0.69, respectively. There was no difference in the proportion of patients with predicted favourable anatomy declining surgery based on the mechanism of IF (25.3% of SBS patients vs. 18.4% fistula patients, *p* = 0.49) or disease leading to IF (32.1% of Crohn’s disease patients vs. 20.5% surgical complication patients vs. 21.7% mesenteric ischaemia patients, *p* = 0.46). Patients with EAF were more likely to decline surgery in comparison to patients with ECF although the result was not statistically significant (30.0% vs. 10.3%, *p* = 0.17).

### 3.4. Nutritional Autonomy

#### 3.4.1. Overall Achievement of Nutritional Autonomy in Entire Cohort

**Figure 2:**
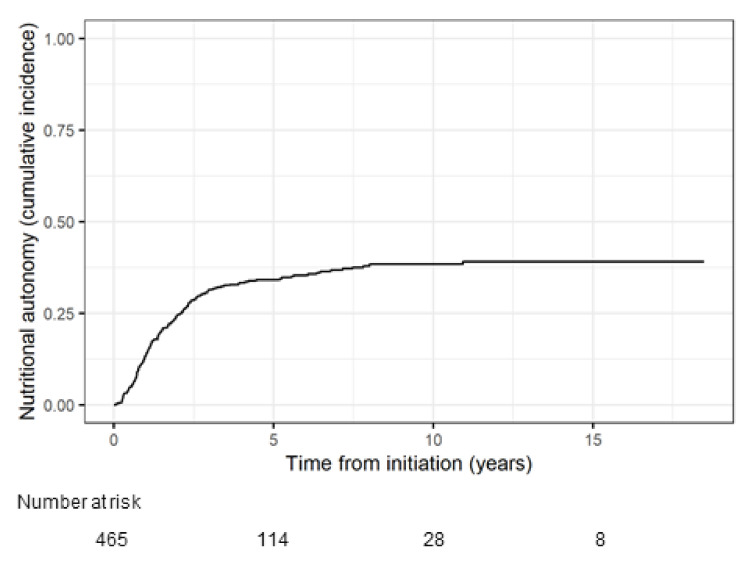
Cumulative incidence function of achieving nutritional autonomy.

Median duration of HPN for all patients included in the analysis was 2.6 years (IQR 1.2–4.9, range 0.04–18.5), with a total time on HPN of 1704.8 patient years. Overall, 170 patients achieved nutritional autonomy. The probability of achieving nutritional autonomy was 13.8% at 1 year, 24.5% at 2 years, 34.1% at 5 years and 38.3% at 10 years (Figure 2).

**Figure 3:**
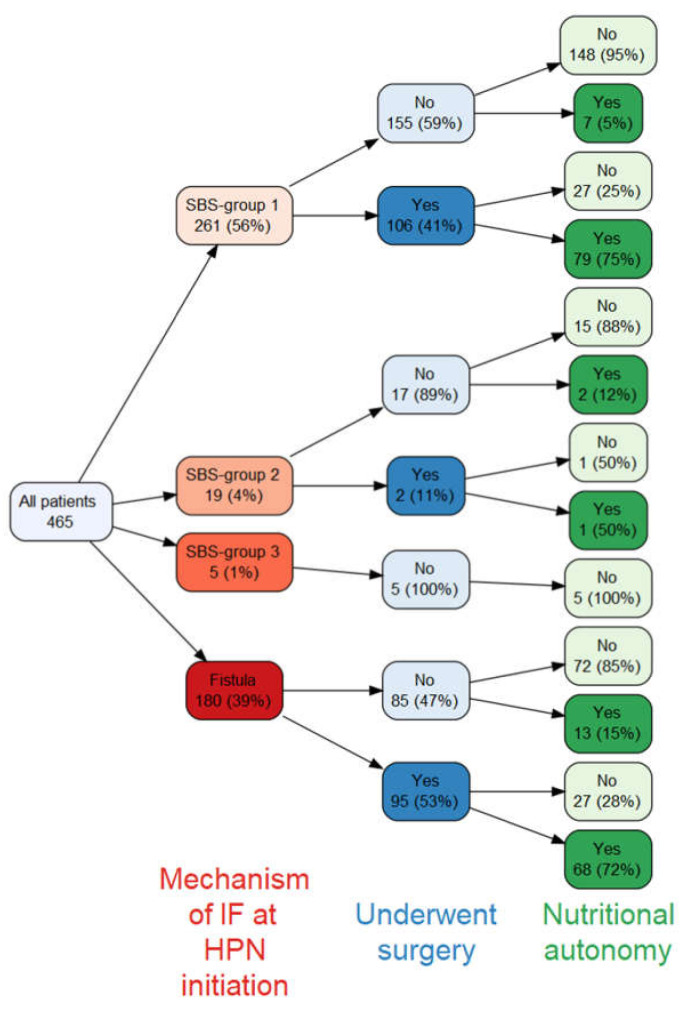
Relationship between mechanism of IF at HPN initiation, subsequent surgery and achieving nutritional autonomy. HPN, Home Parenteral Nutrition.

The relationship between the mechanism of IF at HPN initiation, surgery and nutritional autonomy is shown in Figure 3. Similar proportions of patients with SBS group 1 and intestinal fistulas underwent surgery (41% vs. 53%, respectively) and out of those who did, similar proportions achieved nutritional autonomy (75% vs. 72%, respectively). Of the 13 patients with fistula who achieved nutritional autonomy without undergoing surgery, 7 had spontaneous closure of the fistula.

A further Fine and Gray regression was performed to identify predictors of achieving nutritional autonomy. Younger age, greater small bowel length and presence of colon in continuity were all associated with an increased likelihood of achieving nutritional autonomy (Table 2).

#### 3.4.2. Nutritional Autonomy after Surgery

The rate of achieving nutritional autonomy was higher in patients who underwent reconstructive surgery (109.8 incidences per 1000 patient years) in comparison to patients who did not undergo surgery (18.1 incidences per 1000; *p* < 0.001). The median time of achieving nutritional autonomy after surgery was 5.2 months (95% CI 4.4–7.1), which was significantly affected by the final bowel length (*p* < 0.001) (Supplementary Materials Table S2). There was no significant difference in time to achieve nutritional autonomy for SBS patients in comparison to fistula patients (median 5.2 months vs. 4.8 months, respectively, *p* = 0.50).

### 3.5. Outcomes in Those Predicted to Achieve Nutritional Autonomy Based on Final Intestinal Anatomy

**Figure 4:**
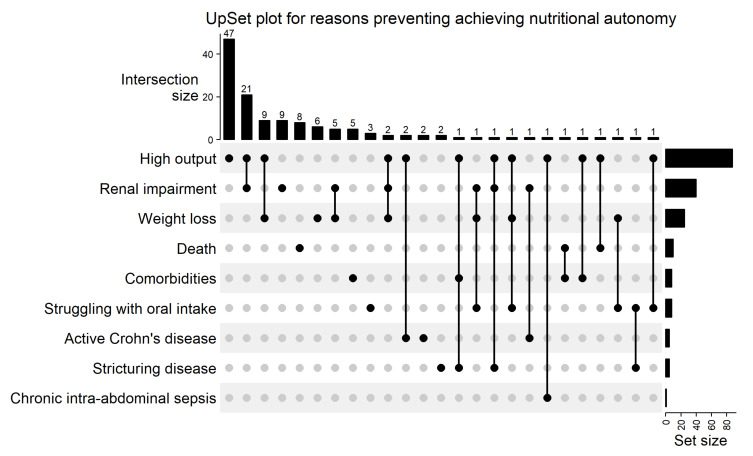
An UpSet plot to illustrate the distribution of reasons for not achieving nutritional autonomy in patients predicted to do so. The x-axis shows possible reason combinations. Each filled-in node shows an identified reason, with the vertical lines linking each reason within the combination. The frequency of each reason combination is shown along the y-axis, correlating to the number of patients with identified reasons as shown by the filled-in nodes. Set size represents the overall frequency of each reason.

A sub-analysis of patients who were “predicted” to achieve nutritional autonomy based on their final intestinal anatomy was performed. Of the 295 patients predicted to achieve autonomy only 162 (54.9%) achieved it. There was a significant difference in achieving nutritional autonomy for different anatomy groups, with only 29.1% of patients with ≥115 cm of small bowel to end-ostomy or fistula (SBS group 1) achieving nutritional autonomy in comparison to 73.1% of patients with small bowel length of ≥60 cm anastomosed to >50% or <50% colon (SBS group 2) and 78.9% of patients with small bowel length of ≥35 cm anastomosed to intact colon (SBS group 3), (*p* < 0.001). Of note, 8 (4.7%) patients who were not predicted to achieve autonomy based on their anatomy were successful in ceasing HPN. The most common reason for not achieving autonomy was high volume output (from stoma, fistula or diarrhoea), followed by renal impairment and weight loss. Each patient could have had one or more reasons for not achieving autonomy, which are presented in Figure 4.

Patients who were predicted to achieve autonomy and did not were significantly older at HPN initiation than the cohort who achieved autonomy (median age 59.0 vs. 49.0, *p* < 0.001), had higher Charlson Comorbidity Index (median score 3 vs. 1, *p* < 0.001), were less likely to undergo reconstructive surgery (9.3% vs. 72.2%, *p* < 0.001) and received HPN for a longer period of time prior to surgery (median 18.9 months vs. 9.7 months, *p* = 0.043) (Supplementary Materials Table S3).

### 3.6. Survival

Overall, by the censoring date, 215 out of 465 (46.2%) patients died. There was no difference in the mortality rates between patient with SBS in comparison to patients with fistulas (81.4 deaths per 1000 person years vs. 87.8 deaths per 1000 person years, respectively) and between ECF and EAF (93.5 deaths per 1000 person years vs. 78.7 deaths per 1000 person years, respectively). The cause of death was IF related in 23 out of 215 (10.7%) patients, underlying disease in 43 (20.0%), other in 130 (60.5%) and unknown in 19 (8.8%) patients. Regarding IF-related causes, 6 patients died due to catheter-related bloodstream infection, 15 due to IFALD and 2 after IF surgery (one after small bowel transplant and one after reconstructive surgery).

In total, 62/203 (30.5%) of the patients who underwent surgery died. The 30-day survival post-surgery was 99.0% and 1-year survival was 94.4%. The cause of death after surgery was IF related in 7 (11.3%) patients (IFALD in 5 patients and following surgery in 2 patients as above), underlying disease in 12 (19.4%), other in 29 (46.7%) and unknown in 14 (22.6%). The median time of death after surgery in patients with unknown causes of death was 9 years with earliest death occurring 3.7 years after surgery.

## 4. Discussion

This is one of the largest and longest single-centre series reporting outcomes in patients with SBS, ECFs or EAFs associated with intestinal failure and the first one to specifically compare outcomes of SBS to intestinal fistulas. The probabilities of achieving nutritional autonomy in our cohort were 13.8% at 1 year, 24.5% at 2 years, 34.1% at 5 years and 38.3% at 10 years, which is similar to previous studies focusing on outcomes in SBS [5,14,15,16]. Younger age, greater small bowel length and presence of colon in continuity were all associated with an increased likelihood of achieving nutritional autonomy. The novelty of our study was the inclusion of a relatively large number of patients with intestinal fistulas over a very long follow-up period, in addition to those with SBS, and our demonstration that there are no differences in long-term nutritional or survival outcomes between patients with SBS and intestinal fistulas nor between patients with ECF and EAF. There were also no significant differences in the proportion of patients undergoing surgery, time to surgery and regaining nutritional autonomy after surgery between patients with SBS and intestinal fistulas. Furthermore, this is the first study to describe reasons for not undergoing surgery in patients with favourable gastrointestinal anatomy, with frailty being the most common but a notable proportion of people also declining surgery despite their HPN dependency. Our study also demonstrates that a substantial proportion of patients who had been predicted to cease HPN based on gastrointestinal anatomy and small bowel length failing to do so, particularly in the SBS group 1 and those of older age with more comorbidity.

Once IF has developed, a structured, multidisciplinary approach is essential for reducing morbidity and mortality as well as restoring nutritional autonomy. In the acute setting of type 2 IF, this includes resolving sepsis, optimising nutritional status, defining intestinal anatomy and then formulating a definitive management plan, which may include reconstructive surgery if appropriate after a period of recuperation at home on parenteral nutrition [2]. The primary aim of reconstructive surgery is to increase the length of the small bowel and, where possible, bring the colon into continuity in order to reduce or obviate patients’ dependency on HPN and improve their quality of life [17,18]. It has been shown that this strategy not only has direct benefits by increasing the available absorptive area, it also has indirect effects related to promoting further intestinal adaptation through trophic effects on the proximal intestine as well as reducing intestinal transit time, possibly as a result of secretion of peptides, including glucagon-like peptide 2 (GLP-2) [4,19]. In the present study, reconstructive intestinal surgery significantly facilitated HPN weaning in patients with severe IF, which is in keeping with previous studies [15,20,21]. While undertaking surgical intervention too early might put the patient at risk of further injury and complication, and a period of 6 months has been recommended before undertaking reconstructive surgery in patients with intestinal fistula, our study identified an even longer time period between initiation of HPN and reconstructive surgery, which also lengthened during the study period. This could be because the older and more comorbid patients identified in the later study periods required more time for preoperative optimisation. Furthermore, our unit has developed an increasing focus on psychological wellbeing prior to any reconstructive surgery in recent years, and it would be useful to explore whether tailored engagement with our psychology team has had a contributing impact.

Although recommendations regarding optimal clinical characteristics for any surgery have been evaluated in the past [1,4,22,23], this is the first study to explore the reasons for patients with potentially favourable predicted gastro-intestinal anatomy not undergoing surgery. While almost half of these patients could not undergo surgery due to comorbidities, frailty or unsatisfactory cardiopulmonary exercise testing (CPET) assessment, it is notable that a significant proportion (22.7%) actually chose not to undergo surgery, despite having been assessed to be fit enough to do so and potentially having an adequate bowel length post-surgery to reduce HPN requirements. We found that, while the decision to decline surgery was not associated with the disease leading to IF, patients with EAF were more likely to decline further surgical management in comparison to those with ECF. It has been recognised that complex abdominal wall reconstruction for EAF is more challenging and potentially more disabling, which could have contributed to these decisions. These data show that despite HPN being burdensome on the quality of life [18,24,25], a significant proportion of patients seem to find it tolerable and a more desired option than surgery. Nonetheless, the comparable long-term nutritional and survival outcomes between those with intestinal fistula and SBS that have now been identified in this study will clearly be of prognostic importance, particularly to those with EAF or ECF, and will help to inform surgeons and patients in their decisions.

Another novel finding of our study was that 45.1% of patients predicted to achieve autonomy based on their final gastrointestinal anatomy failed to do so. This is substantially more than described in previous studies with rates around 12% in a study by Nightingale et al. [26] and 17% by Messing et al. [5]. This discrepancy could be explained by the differences in patient populations as the previous studies included significantly higher proportions of patients with SBS groups 2 and 3 in comparison to group 1 and did not include intestinal fistulas. In addition, patient age in our study was higher with a median of 57 years in comparison to 51 and 52 years in the previous studies. In our cohort, the most common reasons for not achieving autonomy were high volume output, renal impairment and weight loss in patients who were significantly older, had higher Charlson Comorbidity Index, and those who received HPN for a longer period of time prior to surgery. This is consistent with findings in animal studies showing that the adaptive response of the gut is impaired in ageing [27], and our study is the first to demonstrate that the same may be the case in humans. Cell dynamics such as cell turnover rate, crypt cell production rates and enterocyte migration rates are important determinants in the process of intestinal adaptation. Ageing has a significant impact on these processes and results in a higher proliferative rate of crypt cells, possibly leading to a reduction in the number of transporting enterocytes and so reducing nutrient absorption [8,9]. Our results may therefore better inform patient expectation and planning of alternative therapies such as small bowel lengthening [28] or treatment with entero-hormones such as (GLP-2 analogues) [29]. This is especially important for SBS group 1 where nutritional and fluid needs are unlikely to change with time as so far there is limited evidence for any structural or functional adaptation in this patient cohort without treatment [30]. Moreover, our data highlight that the prediction of achieving autonomy is less accurate in patients with SBS group 1 in comparison to groups 2 and 3, which could be explained by the small numbers of patients with SBS group 1 that previous studies have included in the development of previously established bowel length cut-off values [1,5,6,15,23]. Thus, the development of more robust models taking other patient factors in addition to small bowel length in a large patient population would be beneficial for future studies.

The main limitation of the study is that medical records were used as a data collection source. However, the patient database was maintained prospectively and all the patient records were accessible electronically, such that the only missing data involved gastro-intestinal anatomy for 16 (3.4%) cases. In addition, the single-centre nature of the study may limit generalisability. However, this clearly allowed for consistent data collection from a very large cohort over a long period in a national referral centre that may be difficult to replicate elsewhere; this may also have reduced variation in clinical practice affecting outcomes since the data derive from care delivered by an experienced multi-disciplinary team. In addition, due to irretrievable pharmacy prescription data for patients before 2011, we have not collected specific data on patient medications in this study. Lastly, frailty was included as a reason preventing surgical procedure if it was specifically stated in clinical records; however, we did not utilise a specific tool to define its severity, which would be an important next step in future studies.

## 5. Conclusions

In conclusion, we demonstrate one of the largest single-centre experiences describing the long-term ability of HPN-dependent patients with SBS or intestinal fistula to achieve nutritional autonomy. Reconstructive surgery facilitates HPN cessation but the reasons for patients not undergoing surgery are complex. Our data can inform the development of models aimed at predicting nutritional autonomy that will better inform patient expectation and help the planning of alternative therapies.

## Figures and Tables

**Table 1:** Patient demographics and clinical characteristics at HPN initiation. Severity of IF was classified according to ESPEN guidelines [11].

Characteristics		Total (*n* = 465)
Age	Median (IQR)	57.0 (21.0)
Sex (*n*/%)	Male	222 (47.7)
	Female	243 (52.3)
Charlson Comorbidity Index	Median (IQR)	2.0 (3.0)
Mechanism of IF (*n*/%)	SBS group 1	261 (56.1)
	SBS group 2	19 (4.1)
	SBS group 3	5 (1.1)
	Fistula	180 (38.7)
Diagnosis leading to IF (*n*/%)	Surgical complications	198 (42.6)
	Crohn’s disease	118 (25.4)
	Mesenteric ischemia	107 (23.0)
	Volvulus	11 (2.4)
	Trauma	6 (1.3)
	Radiation enteritis	5 (1.1)
	Other	20 (4.3)
Severity of IF (*n*/%)	FE1	18 (3.9)
	FE2	23 (4.9)
	FE3	6 (1.3)
	FE4	2 (0.4)
	PN1	15 (3.2)
	PN2	175 (37.6)
	PN3	144 (31.0)
	PN4	36 (7.7)
	(Missing)	46 (9.9)
Number of nights on HPN per week (*n*/%)	≤3	2 (0.4)
	4 to 5	54 (11.6)
	6 to 7	407 (87.5)
	(Missing)	2 (0.4)

IF, intestinal failure; SBS, Short bowel syndrome; FE, Fluids and electrolytes; PN, Parenteral nutrition; HPN, Home Parenteral Nutrition; IQR, Interquartile range.

**Table 2:** Fine and Gray regression of nutritional autonomy.

Characteristics		SHR (95% CI, *p* Value)
Sex	Male	Reference
	Female	1.16 (0.83 to 1.62, *p* = 0.60)
Age	Median (IQR)	0.98 (0.97 to 0.99, *p* = 0.003)
Underlying disease	Crohn’s disease	Reference
	Mesenteric ischemia	1.38 (0.85 to 2.23, *p* = 0.19)
	Surgical complications	0.99 (0.65 to 1.50, *p* = 0.97)
Final small bowel length in continuity	<50 cm	Reference
	50–99 cm	5.49 (1.31 to 22.98, *p* = 0.02)
	100–149 cm	14.61 (3.61 to 59.14, *p* < 0.001)
	150–200 cm	19.18 (4.80 to 76.72, *p* < 0.001)
	>200 cm	36.09 (8.98 to 145.07, *p* < 0.001)
Final digestive circuit	Colon not in continuity /No colon remaining	Reference
	Colon in continuity and <50% remaining	3.67 (1.12 to 12.01, *p* = 0.032)
	Colon in continuity and >50% remaining	5.91 (3.89 to 8.95, *p* < 0.001)

SHR, Sub-distribution Hazard Ratio; CI, Confidence Interval.

## Data Availability

The data presented in this study are available on request from the corresponding author.

## Supplementary Materials

The following supporting information can be downloaded at: https://www.mdpi.com/article/10.3390/nu14071449/s1, Figure S1: Patient Flow Chart; Table S1: Characteristics of the intestinal tract; Table S2: Median time to achieve nutritional autonomy from the surgery date in patients who underwent reconstructive surgery; Table S3: Comparison of patient characteristics for patients predicted to achieve autonomy who achieved it and did not achieve it.
