# Supplementary material for: Long-Term Outcomes in Patients with Intestinal Failure Due to Short Bowel Syndrome and Intestinal Fistula
Source: Nutrients. 2022 Mar 30;14(7):1449. doi: 10.3390/nu14071449 (PMC9003376; doi:10.3390/nu14071449)
Supplement: Supplementary file 1 [file nutrients-14-01449-s001.zip › nutrients-1652422-supplementary.pdf]

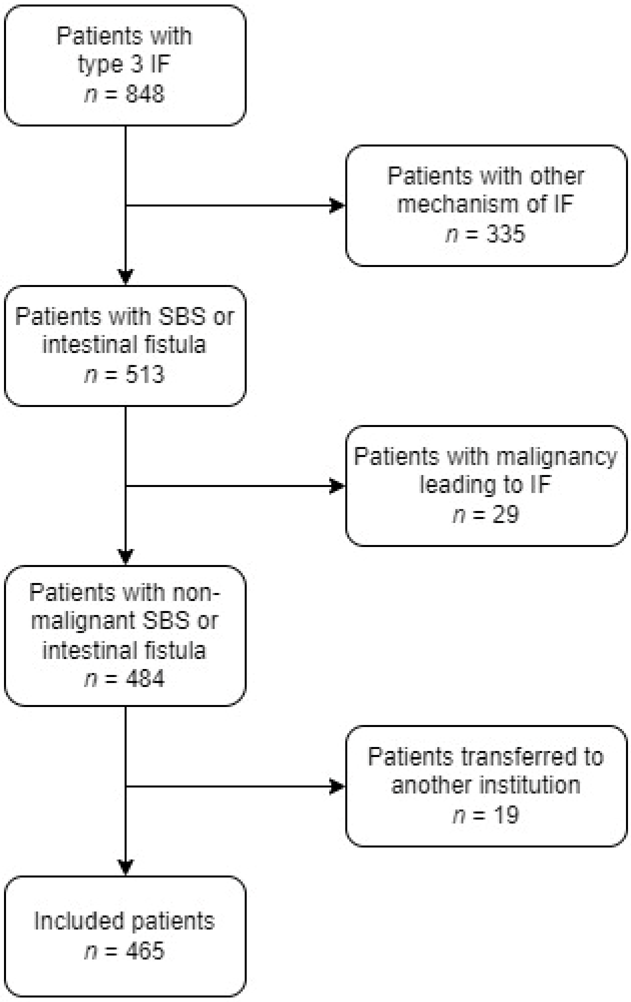

Figure S1. Patient Flow Chart. IF, Intestinal failure; SBS, Short bowel syndrome.

Table S1. Characteristics of the intestinal tract.

Anatomy		Total
Initial small bowel length in continuity (*n*/%)	<50cm	102 (21.9)
	50–99cm	119 (25.6)
	100–149cm	94 (20.2)
	150–200cm	129 (27.7)
	(Missing)	21 (4.5)
Initial small bowel length in continuity (cm)	Median (IQR)	90.0 (100.0)
Initial total small bowel length remaining (in and out of continuity; cm)	Median (IQR)	160.0 (150.0)
Initial colon in continuity (*n*/%)	No	441 (94.8)
	Yes	24 (5.2)
Initial colon length (*n*/%)	<50%	74 (16.2)
	>50%	309 (67.6)
	No colon	74 (16.2)
Final small bowel length in continuity (*n*/%)	<50cm	63 (13.5)
	50–99cm	89 (19.1)
	100–149cm	83 (17.8)
	150–200cm	113 (24.3)
	>200cm	101 (21.7)
	(Missing)	16 (3.4)
Final small bowel length in continuity (cm)	Median (IQR)	140.0 (130.0)
Final total small bowel length remaining (in and out of continuity; cm)	Median (IQR)	160.0 (140.0)
Final colon in continuity (*n*/%)	No	280 (60.2)
	Yes	185 (39.8)
Final colon length (*n*/%)	<50%	75 (16.4)
	>50%	304 (66.5)
	No colon	78 (17.1)
Final presence of stoma (*n*/%)	No	166 (35.7)
	Yes	290 (62.4)
	(Missing)	9 (1.9)

IQR, Interquartile range.

Table S2. Median time to achieve nutritional autonomy from the surgery date in patients who underwent reconstructive surgery.

Final small bowel length	*n*	Median time (months)	95% CI
<50cm	3	41.9	27.1 to NA
50–99cm	12	15.4	12.8 to 39.1
100–149cm	23	5.2	3.5 to 7.8
150–200cm	43	4.8	4.1 to 6.9
>200cm	65	2.3	0.8 to 4.4

CI, Confidence Interval; NA, not applicable—upper 95% CI for the <50 cm group was not able to be calculated due to a small group size.

Table S3. Comparison of patient characteristics for patients predicted to achieve autonomy who achieved it and did not achieve it.

Characteristics		Not achieved autonomy	Achieved autonomy	Total	*p* value
Age	Median (IQR)	59.0 (18.0)	49.0 (26.8)	56.0 (24.0)	<0.001
Sex (*n*/%)	Male	70 (52.6)	81 (50.0)	151 (51.2)	0.739
	Female	63 (47.4)	81 (50.0)	144 (48.8)	
Charlson Comorbidity Index	Median (IQR)	3.0 (3.0)	1.0 (2.0)	2.0 (3.0)	<0.001
Mechanism of IF (*n*/%)	Fistula	50 (37.6)	76 (46.9)	126 (42.7)	0.136
	SBS	83 (62.4)	86 (53.1)	169 (57.3)	
Underlying disease (*n*/%)	Crohn's disease	37 (27.8)	44 (27.2)	81 (27.5)	0.265
	Mesenteric ischemia	19 (14.3)	33 (20.4)	52 (17.6)	
	Surgical complications	72 (54.1)	73 (45.1)	145 (49.2)	
Severity of IF (*n*/%)	FE1	5 (3.8)	12 (7.4)	17 (5.8)	0.237
	FE2	9 (6.8)	4 (2.5)	13 (4.4)	
	FE3	11 (8.3)	7 (4.3)	18 (6.1)	
	FE4	4 (3.0)	1 (0.6)	5 (1.7)	
	PN1	1 (0.8)	1 (0.6)	2 (0.7)	
	PN2	5 (3.8)	8 (4.9)	13 (4.4)	
	PN3	53 (39.8)	56 (34.6)	109 (36.9)	
	PN4	34 (25.6)	54 (33.3)	88 (29.8)	
	(Missing)	8 (6.0)	10 (6.2)	18 (6.1)	
Reconstructive surgery (*n*/%)	No	7.0 (2.0)	7.0 (2.0)	7.0 (2.0)	<0.001
	Yes	96 (72.2)	15 (9.3)	111 (37.6)	
Length of HPN prior to surgery (months)	Median (IQR)	37 (27.8)	147 (90.7)	184 (62.4)	0.043
		18.9 (17.4)	9.7 (14.8)	11.4 (15.5)	

Severity of IF was classified according to ESPEN guidelines [11]. IF, Intestinal failure; SBS, Short bowel syndrome; FE, Fluids and electrolytes; PN, Parenteral nutrition; HPN, Home Parenteral Nutrition.
